# The Novel Oomycide Oxathiapiprolin Inhibits All Stages in the Asexual Life Cycle of *Pseudoperonospora cubensis* - Causal Agent of Cucurbit Downy Mildew

**DOI:** 10.1371/journal.pone.0140015

**Published:** 2015-10-09

**Authors:** Yigal Cohen

**Affiliations:** The Mina & Everard Goodman Faculty of Life Sciences, Bar-Ilan University, Ramat-Gan, 5290002, Israel; University of Nebraska-Lincoln, UNITED STATES

## Abstract

Oxathiapiprolin is a new oomycide (piperidinyl thiazole isoxazoline class) discovered by DuPont which controls diseases caused by oomycete plant pathogens. It binds in the oxysterol-binding protein domain of Oomycetes. Growth chambers studies with detached leaves and potted plants showed remarkable activity of oxathiapiprolin against *Pseudoperonospora cubensis* in cucurbits. The compound affected all stages in the asexual life cycle of the pathogen. It inhibited zoospore release, cystospore germination, lesion formation, lesion expansion, sporangiophore development and sporangial production. When applied to the foliage as a preventive spray no lesions developed due to inhibition of zoospore release and cystospore germination, and when applied curatively, at one or two days after inoculation, small restricted lesions developed but no sporulation occurred. When applied later to mature lesions, sporulation was strongly inhibited. Oxathiapiprolin suppressed sporulation of *P*. *cubensis* in naturally-infected leaves. It exhibited trans-laminar activity, translocated acropetaly from older to younger leaves, and moved from the root system to the foliage. Seed coating was highly effective in protecting the developed cucumber plants against downy mildew. UV microscopy observations made with cucumber leaves infected with *P*. *cubensis* revealed that inhibition of mycelium growth and sporulation induced by oxathiapiprolin was associated with callose encasement of the haustoria.

## Introduction

Downy mildew caused by *Pseudoperonospora cubensis* is a major disease of cucurbits. It attacks cucumber, melon, squash, pumpkin and various gourds [1,2]. The pathogen has undergone major changes during the last decade. Disease severity and epidemics became far more destructive and new genotypes made disease control through host plant resistance ineffective. Consequently, disease control relies mainly on oomycides applications [3,4]. Sixteen chemical classes with different modes of action are available for the control oomycete plant pathogens (www.frac.info). The three most important single-site compounds are Phenylamides (PAs), Quinone outside Inhibitors (QoIs) and Carboxylic Acid Amides (CAAs) [5].

Resistance to single-site oomycides has developed in many oomycete plant pathogens including *P*. *cubensis*. Isolates resistant to mefenoxam and strobilurins [6] were found in US fields [7,8]. Resistance was also reported for cymoxanil and propamocarb [9,10].

Resistance to PAs in *P*. *cubensis* was discovered in Israel two years after introduction of metalaxyl [11] and thereafter in other countries [10,12,13]. PA-resistant isolates in Israel possessed higher competitive fitness [14] and exhibited much reduced sensitivity to mancozeb compared to PA-sensitive isolates [15]. They remained predominant in the population after metalaxyl was abandoned [16]. Most *P*. *cubensis* isolates collected during 2008–2011 in Israel and the USA were resistant to PA [17]. Pathotype 6 is a new comer in Israel [14]; resistance to PA is common on cucumber and melon attacked by pathotype 3, but rare on squash, pumpkin and butternut gourd which are attacked by pathotype 6 (Y. Cohen, A.E. Rubin and L. Falach, *unpublished data*). Resistance to PA was found among isolates of *P*. *cubensis* collected in China in 2012, Vietnam in 2013 and India in 2014 [3]. Varying frequencies of PA resistant isolates were detected in the Czech Republic between 2005 and 2009 [10,13].

QoI resistant *P*. *cubensis* isolates (carrying G143A mutation) were detected in Japan by Ishii et al. [18]. High frequencies of QoI resistance occurs in Israel and many other locations [13], especially in the East Coast of the USA (QoI FRAC working group minutes, 2014, www.frac.info).

Resistance to CAAs in *P*. *cubensis* is associated with two different amino acid exchanges in its target protein, CesA3: at position G1105V (glycine for valine, in Israeli isolates) and at position G1105W (glycine for tryptophan, in USA isolates) [19,20]. Both mutations confer cross resistance among CAAs [20]. Resistance of *P*. *cubensis* to CAAs was reported in the West Coast of the USA, Israel and China (CAA FRAC working group, www.frac.info; [21]. Isolates collected in Vietnam and Russia were all sensitive to CAAs (Y. Cohen, *unpublished data*).

The build-up of resistances to single-site oomycides has accelerated the search for anti-oomycete compounds with new modes of action. Two such novel compounds became recently available: ametoctradin (Quinone QoSI inhibitor of the respiratory chain) binding to the mitochondrial bc1 complex III [22–24], and oxathiapiprolin (inhibitor of oxysterol-binding protein) [25]. Sweigard et al. [25] reported on identification of the oxysterol-binding protein (OSBP) as the target protein of this oomycide. Recent studies showed that oxathiapiprolin is effective against downy mildew in basil caused *Peronospora belbahrii* [26,27], black shank in tobacco caused by *Phytophthora nicotianae* [28], *P*. *capsici* in bell pepper [29–31] and downy mildew in sunflower caused by *Plasmopara halstedii* [32]. No studies are available on the activity of oxathiapiprolin against other oomycetes including *P*. *cubensis*.

The purpose of this study was to examine the efficacy of the new anti-oomycete compound oxathiapiprolin (piperidinyl thiazole isoxazoline class) against *P*. *cubensis*. The specific objective was to investigate the effect of oxathiapiprolin on the various stages in the asexual life cycle of *P*. *cubensis*, the agent of downy mildew in *Cucurbitaceae*. A preliminary report on some findings was given before [33].

## Methods and Materials

### Oomycide

Oxathiapiprolin (DPX-QGU42-10OD, 10% active ingredient formulated as oil dispersion) was obtained from DuPont De Numerous. It was suspended in water to produce a stock suspension of 1mg/ml (1000 mg/l). Tenfold serial dilution was made to prepare 0.0001 to 100 mg/l suspensions. All concentrations are given as mg/l active ingredient (a.i.).

### Pathogen

Most experiments were performed with cucumber inoculated with isolate 83c of *P*. *cubensis* (pathotype 3, mating type A1, resistant to mefenoxam, dimethomorph, mandipropamid and azoxysrrobin). The effect of oxathiapiprolin on infection and disease development of cucumber was also tested with additional 25 isolates of the pathogen of various origins, pathotypes, mating types and oomycide-resistance profiles (Table 1). In some experiments, other cucurbit hosts (see below) were used for experiments in the manner described for cucumber. Isolates were maintained by repeated inoculations of detached leaves of cucumber in growth chambers at 15°C (14h light/day). Sporangia were removed from freshly sporulating leaves into distilled water kept on ice, adjusted to 2000 sporangia/ml, and used for inoculation. In some experiments sporangial suspensions were used for *in-vitro* testing of zoospore release, zoospore motility, zoospore encystment and cystospore germination.

**Table 1:** Origin and characteristics of 26 isolates of *Pseudoperonospora cubensis* used in this study.

Number	Isolate	Country	Host	Year	Pathotype^a^	Mating type	Oomycide resistance^b^			
							MFX	MPD	AZX	OXA
1	PCHS	Japan	Unknown	1980	3	A1	S	S	S	S
2	8904	Israel	Cucumber	2004	3	A1	R	S	nt	S
3	19	Israel	Melon	2007	3	A1	R	R	R	S
4	US-163	USA	Watermelon	2008	5	A1	R	R	nt	S
5	42R	Israel	Cucumber	2008	3	A2	R	R	R	S
6	34	Israel	Cucumber	2009	3	A1	R	S	S	S
7	13	Israel	Squash	2010	6	A2	S	S	S	S
8	81	Spain	Cucumber	2011	3	A1	R	R	nt	S
9	83c	Spain	Cucumber	2011	3	A1	R	R	R	S
10	98P	Israel	Pumpkin	2011	6	A2	I	S	S	S
11	101D	Israel	Butter gourd	2011	6	A2	I	S	S	S
12	172S	Israel	Pumpkin	2012	6	A2	R	S	S	S
13	Harbin 10P	China	Cucumber	2012	7	A2	R	R	S	S
14	197c	Israel	Cucumber	2013	3	A1	R	R	R	S
15	BH	Israel	Cucumber	2013	3	A1	R	R	R	S
16	14-RO-06	Romania	Cucumber	2014	3	A1	nt	nt	nt	S
17	14-ES-24	Spain	Cucumber	2014	3	A1	R	S	nt	S
18	14-GR-02	Greece	Cucumber	2014	3	A1	R	S	R	S
19	c3-mpd1000	Israel	Cucumber	2014	3	A1	R	R	R	S
20	c1-azx1000	Israel	Cucumber	2014	3	A1	R	R	R	S
21	Luf-2	India	Luffa	2014	10	A2	S	S	S	S
22	Ash-1	India	Ash gourd	2014	10	A2	S	S	S	S
23	Ger-1	Germany	Pumpkin	2014	6	A2	S	S	S	S
24	227-c	Israel	Cucumber	2015	3	A1	R	R	R	S
25	229P	Israel	Pumpkin	2015	6	A2	S	S	S	S
26	231-c	Israel	Cucumber	2015	3	A1	R	R	R	S

^a^ After Cohen et al, 2015

^b^ MFX = mefenoxam; MPD- mandipropamid; AZ = azoxystrobin; OXA = oxathiapiprolin.

For MFX: S = sensitive, ED50 ≤ 1 mg/l; I = intermediate, ED50 1-10mg/l; R = resistant, ED50 ≥100mg/l.

For MPD and AZX: S = sensitive, ED50 ≤ 1 mg/l; R = resistant, ED50 ≥10mg/l.

For OXA: S = ED50< 0.0001 mg/l.

nt = not tested

### Plants

Cucumber plants (cv. Nadiojni or SMR-18) were used in most experiments. In some experiments, melon (cv. AY), squash (cv. Beruti), pumpkin (cv. Tripoli), butternut gourd (cv. Dalorit), watermelon (cv. Malali), bottle gourd (local cultivar) and ridge gourd (cv. SV) were used. Plants were grown in the greenhouse and used when reached the 2–3 leaf stage (3–4 weeks after sowing). Most assays were conducted with detached leaves, but some were done with whole potted plants or plants grown in net-houses.

### Preventive treatment and inoculation

Detached leaves were placed on moistened filter paper in 14 cm Petri dishes lower surface uppermost (unless stated otherwise). They were sprayed with oxathiapiprolin of various concentrations with the aid of a fine glass atomizer. Leaves sprayed with water served as controls. At 2-3h after spray, leaves were drop inoculated with sporangia of *P*. *cubensis* (400 sporangia per 20 μl, 20 droplets per leaf, unless stated otherwise). Inoculated leaves were incubated in a growth chamber at 20°C (12h light/day, 60 μmole. m^2^.s^-1^) and evaluated for disease development at 7 dpi (days post inoculation), unless stated otherwise.

### Inoculation and curative treatment

Detached leaves were placed on moistened filter paper inside 14 cm Petri dishes lower surface upper-most and drop inoculated with sporangia of *P*. *cubensis* in the manner described above. The inoculated leaves were incubated in a growth chamber at 20°C as above and sprayed at 1–6 (dpi) with water (control) or oxathiapiprolin of various concentrations.

### Sporulation in naturally-infected leaves

Cucumber leaves, naturally-infected with *P*. *cubensis*, were collected at 8 am from Net-house 1 on campus on April 27 and May 1, 2015. Leaves were brought to the laboratory and the sporangia they carried were blown away in a laminar hood with the aid of a strong stream of compressed air. Leaves (n = 4) were laid upper surface uppermost on wet filter in 14 cm Petri dishes and sprayed with water (control) or oxathiapiprolin of various concentrations. Leaves were incubated at 20°C under light (100 μmole.m^2^.s^-1^) until 17 pm, the number of lesions counted (ranged from 120–180 per leaf) and then placed at 18°C in the dark for 16h to allow for sporulation. The sporangia produced were collected with a fine water jet into a beaker, their concentration was determined with the aid of a cytometer and the number of sporangia produced per lesion was calculated.

Similar experiments were conducted with naturally-infected leaves collected on June 9 and June 14, 2015 from Net-house 3 located at Bar-Ilan Farm. In these leaves, downy mildew lesions occupied the whole leaf area. Therefore, sporangial number was determined in 12 mm leaf discs removed from each leaf (n = 3). Leaf discs were placed in 3 ml water containing 0.01% Tween-20, agitated on Vortex (high speed for 1 minute), the number of sporangia was determined with the aid of a cytometer, and the number of sporangia produced per 1cm^2^ leaf tissue was calculated.

The effect of oxathiapiprolin on sporulation of *P*. *cubensis* in naturally-infected cucumber plants was tested directly in Net-house 3. Downy mildew-infected plants (n = 10) were sprayed at 3 weeks after inoculation with oxathiapiroplin (3 mg/l ai) at 8 am 11.6.2015 while control plants (n = 10) were left untreated. Leaves (one per plant) were collected at 8 am 12.6.2015 (night was dewy). Leaves were brought to the laboratory, photographed, and the intensity of sporulation was visually estimated (0–3 scale, with 0 = no visible sporulation; 1 = weak sporulation; 2 = moderate sporulation; and 3 = heavy sporulation).

### Translaminar movement

The following experiments were performed to learn if oxathiapiprolin can translocate from one surface of the leaf to the opposite (thereafter inoculated) surface of that leaf. Detached leaves (n = 3) of cucumber were laid in 14 cm Petri dishes on wet filter paper lower or upper surface uppermost. Leaves were sprayed with oxathiapiprolin of various concentrations and two hours later were drop-inoculated with sporangia (15 droplets, 25 μl each) on the treated surface or on the opposite, untreated surface. Leaves were incubated at 18°C in the dark for 20h to allow for infection and then at 20°C (12h light/day) to enable lesion development. Number and size of lesions was determined at 7 and 14dpi.

### Translocation form leaves or hypocotyl

The following experiments were performed to learn if oxathiapiprolin translocates acropetally from a treated leaf 1 to the newly developed leaf 2. Cucumber plants having one true leaf (n = 3) were either sprayed on that leaf or treated with 10 droplets (25 μl each) of oxathiapiprolin of various concentrations. Control plants were left untreated. Plants were then incubated at 20°C (12h light/day) for 4 days to allow the second true leaf to expand. Plants were then spray-inoculated with the pathogen and disease development on the treated leaf 1 and the untreated leaf 2 was recorded at 7dpi.

In other experiments, oxathiapiprolin was applied as a fine spray to the hypocotyl of horizontally-laid 2-leaf plants (n = 6). To avoid contact with other organs, leaves were covered with a plastic bag and the soil surface covered with aluminum foil. Control plants remained untreated. Plants were inoculated at 6h after treatment and were evaluated for disease development at 7dpi.

### Translocation from the root

Plants (n = 6) at the 2-leaf stage growing in 200 ml pots were irrigated to soil capacity and thereafter drenched around the stem base with 1 ml oxathiapiprolin of 100 mg/l. Plants were thereafter irrigated twice, at 4h intervals, with 5 ml tap water to facilitate the distribution of the oxathiapiprolin through the root system. Plants were incubated at 20°C (12h light/day) and inoculated one day after treatment. Disease records were taken at 7dpi.

### Seed coating

One hundred cucumber seeds (2g) were mixed with 100 mg oxathiapirolin 10%. The mixture was rotated at 200 rpm for 1h and the seeds were allowed to rest on the bench for 10 days. The coated seeds weighed 2.04 g, indicating that each seed was coated with approximately 40 μg active ingredient of oxathiapiprolin. Plants were raised from control and coated seeds and were spray-inoculated *P*. *cubensis* at the 1-leaf 2-leaf or 3- leaf stage. A week later the number of lesions produced was counted on each plant (n = 10).

### *In-vitro* assays

One ml aliquots of sporangial suspensions in 20 ml vials were supplemented with oxathiapiprolin of various concentrations (n = 3), kept on ice for 30 min and then on the bench to allow for zoospore release. Zoospore release and motility were examined in 10 μl droplets at a magnification of x63.

Temporary exposure of sporangia to oxathiapiprolin was tested as follows: sporangia were suspended in water or oxathiapiprolin of various concentrations. After 15 minutes, they were washed with excessive water over 8μ Millipore membrane and re-suspended in fresh water. Zoospore release was examined microscopically at x63 after 2 hours.

Motile zoospores in depression glass slides were amended with oxathiapiprolin of various concentrations (or water) and sucrose to final concentration of 0.1M. Slides were incubated inside moisten Petri dishes at 18°C in the dark. Cystospore germination was examined after 15 h at x63.

### Microscopy

The procedures described by Cohen et al. [34,35] were employed. One cm diameter leaf discs were removed from the test leaves at various time intervals after inoculation or fungicidal treatment. Leaf discs were placed in 5 ml ethanol and boiled for 5 minutes to clarify. The ethanol was discarded and 5 ml of 0.01% aniline blue solution (in 0.1M dibasic-potassium phosphate pH 8.9 buffer) were added. The vials containing the leaf discs were placed at 4°C overnight. The leaf discs were then removed, placed on glass slides, lower surface upper-most, treated with 10μl of 0.01% calcofluor solution (Sigma), covered with a cover slip and examined with aid of Olympus A70 epifluorescent microscope. Electron micrographs were produced with the aid of Phillips SEM after standard processing of the infected tissue.

### Data analysis

Experiments with detached leaves and potted plants were repeated twice. *In vitro* experiments were repeated three times. Results from the repeated experiments showed little variation. Data from repeated experiments were averaged and the mean and standard deviation values were calculated. Analysis of variance was done using JMP^®^ software (SAS Institute). Significant difference between means were calculated for α = 0.05 using Tukey’s-Kramer HSD (honest significant difference) test.

## Results

### Efficacy *in vitro*

#### Zoospore release

Oxathiapiprolin had a strong inhibitory effect on zoospore release from sporangia *in vitro*. The mean number of motile zoospores (±SD) counted per microscope field at x63, at 1.5h after addingoxathiapirolin, was 1400±350, 5.5±6.4, 1.5±2.1, 0.5±0.7, and 0 at oxathiapiprolin concentrations of 0, 0.00001, 0.0001, 0.001 and 0.01 mg/l, respectively.

#### Temporary exposure of sporangia to oxathiapiprolin

Control sporangia in water released a mean of 2000±450 motile zoospores in 10μl suspension. Sporangia continuously exposed to 0.00001 mg/l oxathiapiprolin released 20±30 motile zoospores and sporangia temporarily exposed to 0.00001 mg/l oxathiapiprolin released 200±50 motile zoospores. Exposure to 0.001 mg/l oxathiapiprolin for 15 minutes was totally inhibitory to zoospore release, suggesting that oxathiapiprolin may bind quickly and irreversibly to its target in the pathogen.

#### Effect of oxathiapiprolin on zoospore motility

The oomycide had a weak effect on zoospore motility. At 30 min after adding it to motile zoospore suspension the proportion of halted zoospores was reduced by 25, 50 and 75%, at oxathiapiprolin of 0.0005, 0.05 and 5 mg/l, respectively.

#### Cystospore germination

**Fig 1:**
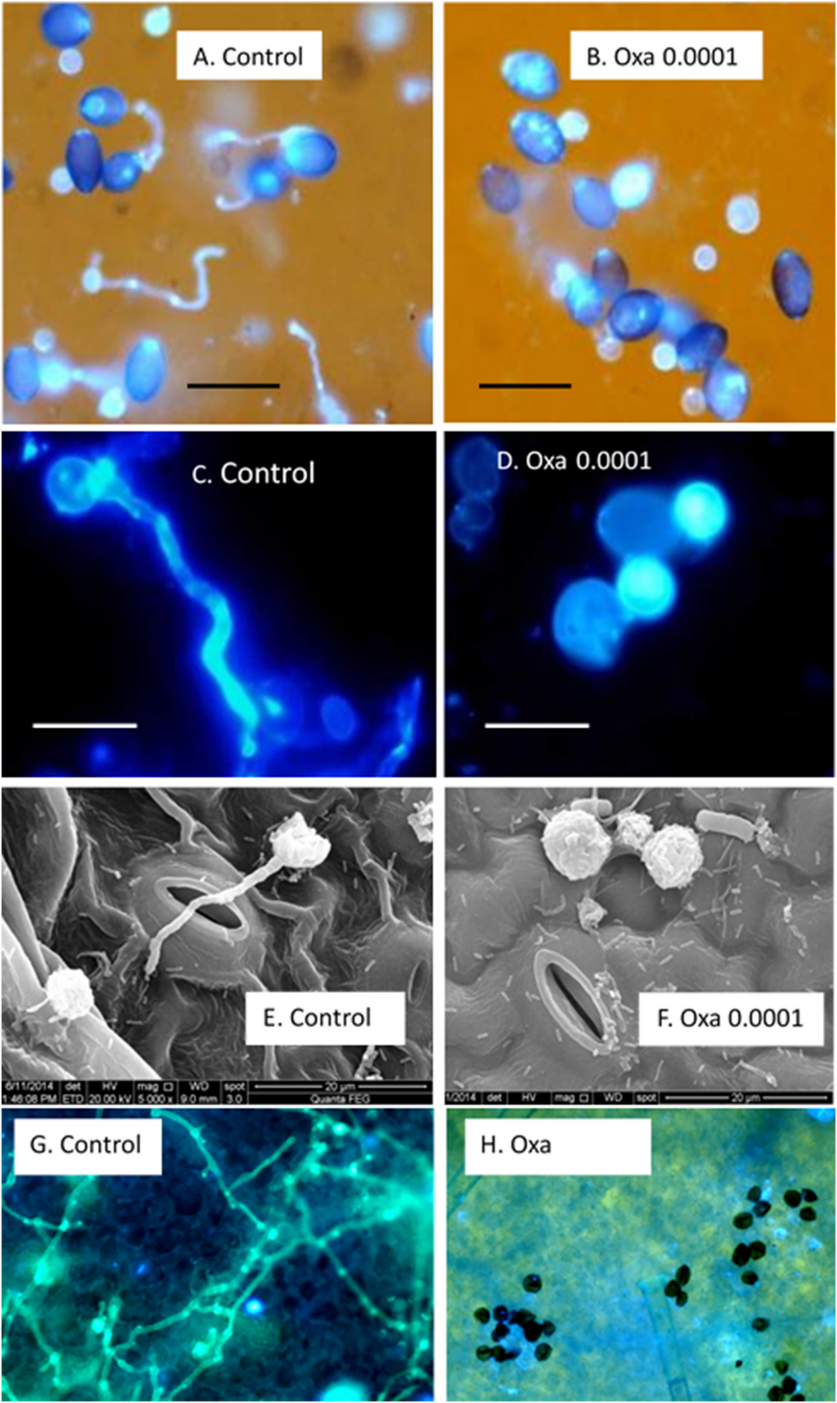
The inhibitory effect of oxathiapiprolin on cystospore germination of *P*. *cubensis*. **A-D** *In vitro*; **E-H** *in vivo*. **A, C**—Fluorescent micrographs (with or without bright light) showing cystospore germination in water. **B**, **D**—No cystospores germination occurred in oxathiapiprolin of 0.0001 mg/l. Bars in **A, B** = 50 μm; in **C, D** = 10 μm. **E**—Scanning electron micrographs showing a germinating cystospore a control leaf. **F**—inhibitory effect of oxathiapiprolin on cystospore germination on a treated leaf. **G**—Fluorescent micrographs showing extensive mycelial growth in a control leaf at 1 dpi. Bar = 100 μm. **H**—Oxathiapiprolin inhibits zoospore discharge on a cucumber leaf surface at 1 dpi. No mycelial growth is seen. Bar = 50 μm.

Oxathiapiprolin had a strong inhibitory effect on cystospore germination *in vitro*. In various experiments, percent germinating cystospores in water amended with 0.1M sucrose ranged from 20 to 30%, as against 0% in oxathiapiprolin of 0.0001 mg/l (Fig 1A–1D).

### Efficacy *in vivo*

#### Zoospore release and cystospore germination

Control-untreated and oxathiapiprolin-treated cucumber leaves were drop-inoculated with sporangia, incubated at 20°C for one or two days and thereafter examined microscopically. Cystospores germinated nicely at 1 dpi on the surface of control untreated inoculated leaves and germ-tubes grew over the stomatal opening (Fig 1A, 1C and 1E). In oxathiapiprolin-treated (0.0001 mg/l) leaves no germinating cystospores were seen (Fig 1B, 1D and 1F). Extensive mycelium growth inside the control leaves was observed at 2 dpi (Fig 1G) while no visible mycelium growth was seen in the treated leaves (Fig 1H) due to the lack of zoospores release and/or cystospore germination.

### Infection and sporulation in detached leaves and potted plants

**Fig 2:**
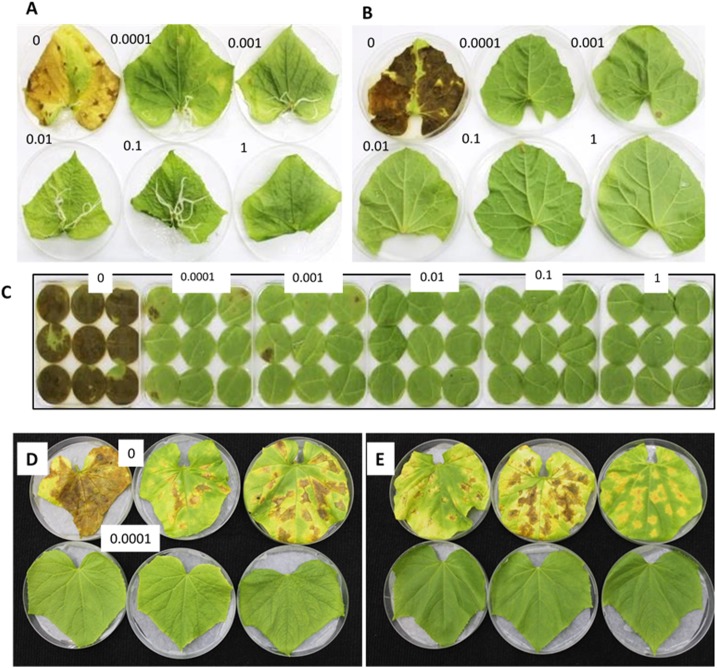
Preventive application of oxathiapiprolin inhibits infection with *P*. *cubensis*. **A**—Detached leaves of cucumber treated with 0.0001–1 mg/l. **B**—Detached leaves of melon treated with 0.0001–1 mg/l. **C**—Leaf discs of melon treated 0.0001–1 mg/l. **D**- Detached leaves of cucumber treated with 0.0001 mg/l and inoculated with three A1 isolates: 19, 42R and BH. **E**- Detached leaves of cucumber treated with 0.0001 mg/l and inoculated with three A2 isolates: 13, 229P and 98P. Leaf tissues were sprayed and inoculated on lower leaf surface. Photos in **A, B** and **C** were taken at 13 dpi with lower surface uppermost; photos in **D** and **E** were taken with upper surface uppermost at 10 dpi.

The effect of a preventive application in detached leaf assays is demonstrated in Fig 2A (cucumber) and Fig 2B (melon). Treatment with oxathiapiprolin of 0.0001–0.001 mg/l totally inhibited disease development, conforming that no penetration has taken place due to the fact that oxathiapiprolin avoided zoospore release and/or cystospore germination. Preventive spray with oxathiapiprolin was highly effective against downy mildew in melon leaf discs. Percent area infected in leaf discs (4 cm diameter) treated with oxathiapiprolin of 0, 0.0001, 0.001, 0.01, 0.1, and 1 mg/l was 97.8±4.4, 4.4±6.8, 2.2±5.1, 0, 0, and 0%, respectively (Fig 2C).

A series of experiment were performed to explore whether isolates of *P*. *cubensis* of various origins and characteristics differ in their sensitivity to oxathiapiprolin. All 26 isolates listed in Table 1, regardless of their origin, pathotype, mating type or sensitivity to other oomycides, produced no lesions when drop-inoculated onto detached cucumber leaves preventively-treated with oxathiapiprolin of 0.0001 mg/l. A1 and A2 isolates were similarly controlled by 0.0001 mg/l of the compound (Fig 2D and 2E). Similarly, the isolates belonging to pathotypes 5, 6, and 10 produced no lesions when drop-inoculated onto detached leaves of their respective hosts watermelon, squash or ridge gourd, respectively, when treated with oxathiapiprolin of 0.0001 mg/l before inoculation.

**Fig 3:**
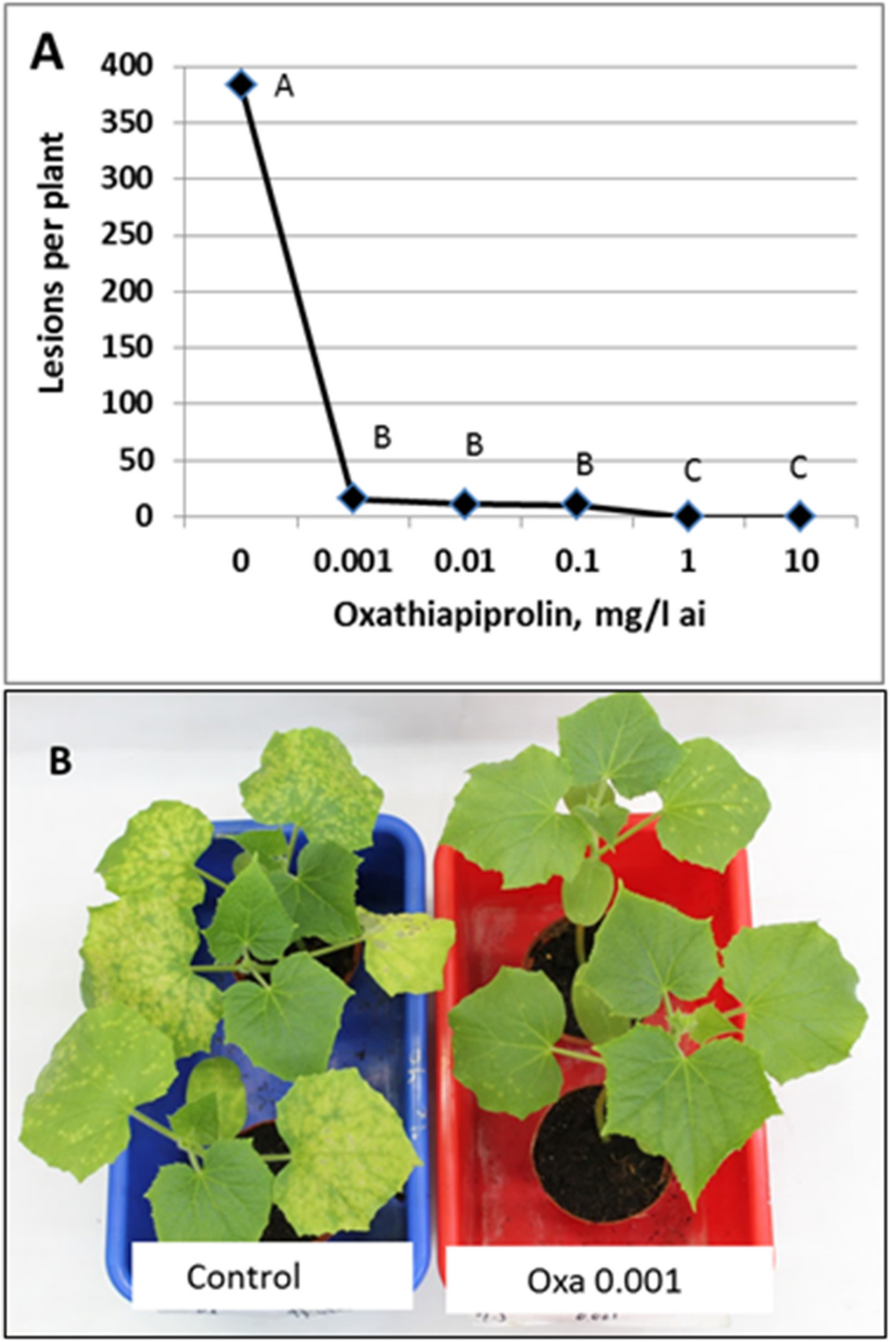
The effect of a preventive spray with oxathiapiprolin on infection of 2-leaf cucumber plants with *P*. *cubensis*. **A**—Mean number of lesions per plant at 7dpi. Different letters on bars indicate on significant differences at α = 0.05 (Tukey’s-Kramer HSD-test). **B**—Disease development at 7 dpi on control and oxathiapirolin treated plants.

In potted plants, the detrimental effect of oxathiapiprolin on infection was significantly expressed at ≥ 0.001 mg/l. Two leaf plants were sprayed with oxathiapiprolin on the upper leaf surface and 3h later were spray-inoculated on the upper leaf surface with sporangia of *P*. *cubensis*. Disease records (taken at 7dpi) showed a mean of 383±76 downy mildew lesions per plant in the controls; this number decreased by 96 and 100%, respectively relative to control, at oxathiapiprolin of 0.001 and 1 mg/l, respectively (Fig 3A and 3B).

**Fig 4:**
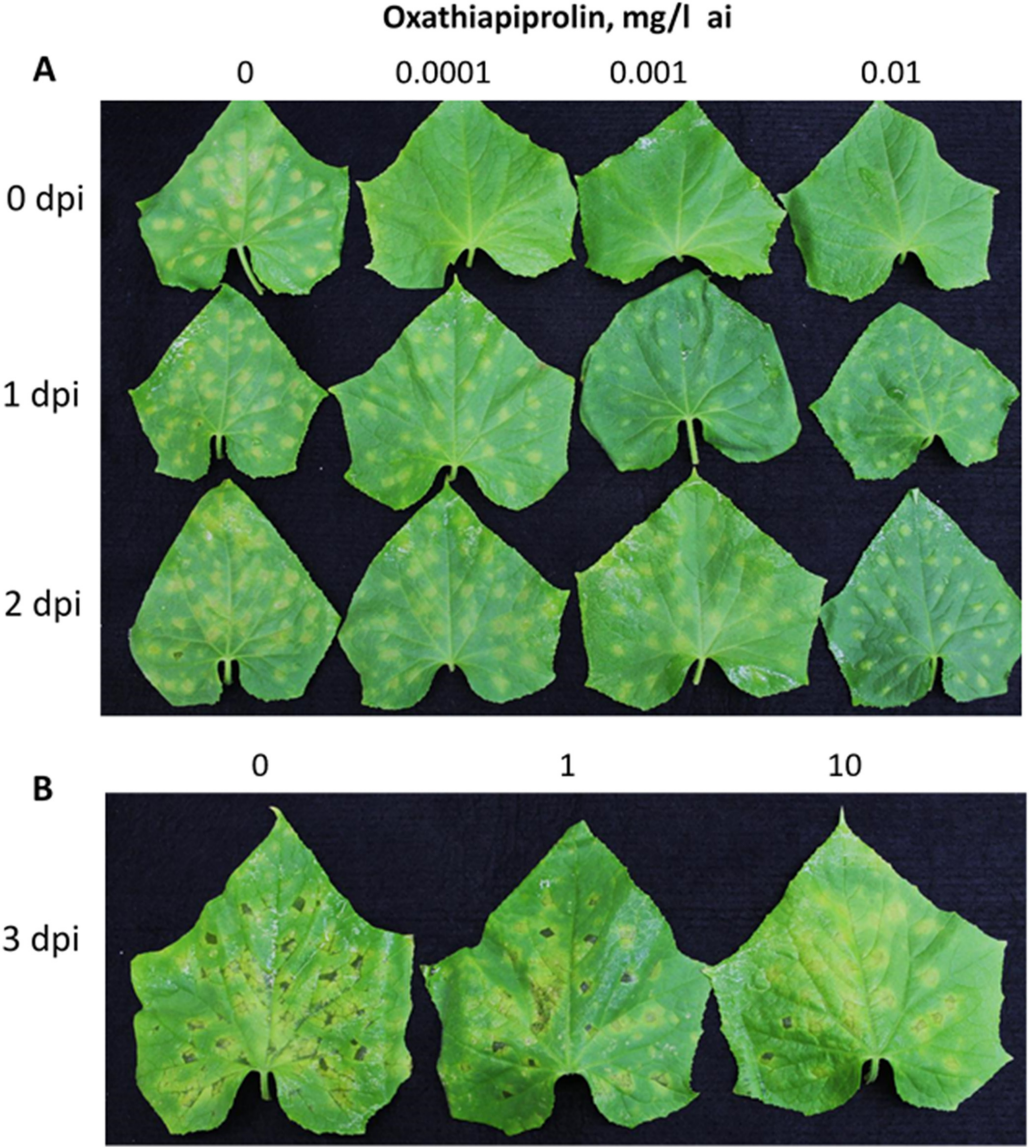
The inhibitory effect of oxathiapiprolin on development of downy mildew in cucumber depends on time of application. Detached leaves were inoculated on lower leaf surface with sporangial suspension (25 droplets/leaf) and oxathiapiprolin was applied as a spray at 0, 1, 2 or 3dpi. **A**—No lesions developed when oxathiapiprolin was applied at 0 dpi. Lesions appeared when oxathiapiprolin was applied at 1 or 2dpi, but became smaller as its dose increased. **B**—Oxathiapiprolin was applied at 3dpi. Note no effect on lesion size. Photos were taken at 6 dpi.

The differential effects of oxathiapiprolin in preventive *vs* curative application is demonstrated in Fig 4. The compound, applied preventively (time zero) at a low dose of 0.0001 mg/l, totally prevented symptom production (Fig 4A upper panel), whereas when applied at 1 or 2dpi, symptoms were produced even at a dose of up to 0.01 mg/l (Fig 4A middle and lower panels). Very slight effect on lesion appearance was seen in leaves treated with oxathiapiprolin at 3dpi, even with a high of 10 mg/l (Fig 4B).

While oxathiapiprolin applied before inoculation suppressed zoospore release and/or cyst germination and therefore inhibited lesion formation, oxathiapiprolin applied after inoculation suppressed lesion expansion, sporangiophore formation and sporangial yield, depending on the time of its application.

**Fig 5:**
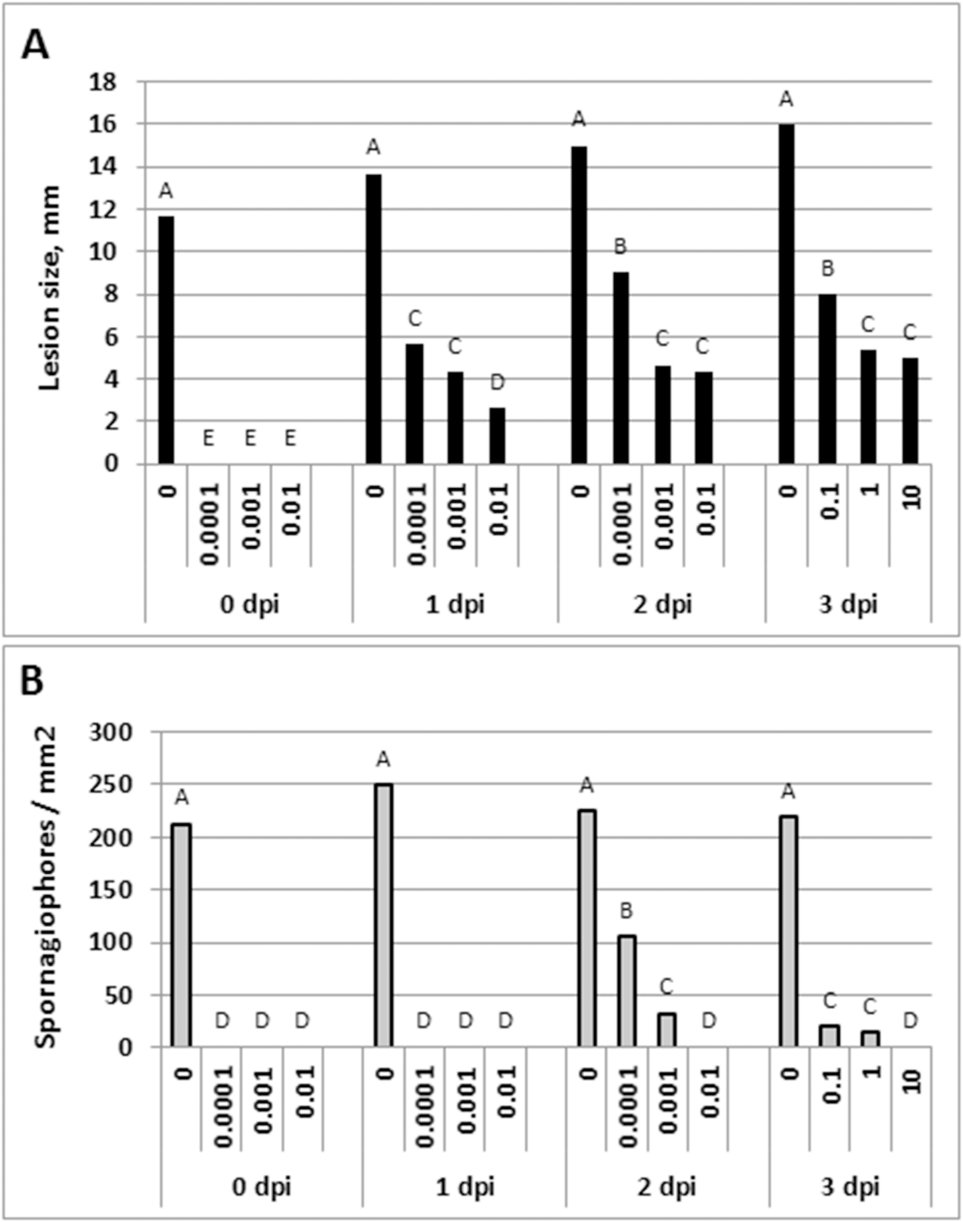
Preventive and curative effects of oxathiapiprolin on lesion development, lesion expansion and sporagiphore formation of *P*. *cubensis* in detached leaves of cucumber. See legend to Fig 4 for experimental design. **A**—Lesion size, as measured at 10 dpi. **B**—Number of sporangiophores produced at 10 dpi per 1 mm^2^ leaf tissue as counted with the aid of a UV epifluorescent microscope. Different letters on bars indicate on significant differences at α = 0.05 (Tukey’s-Kramer HSD-test).

**Fig 6:**
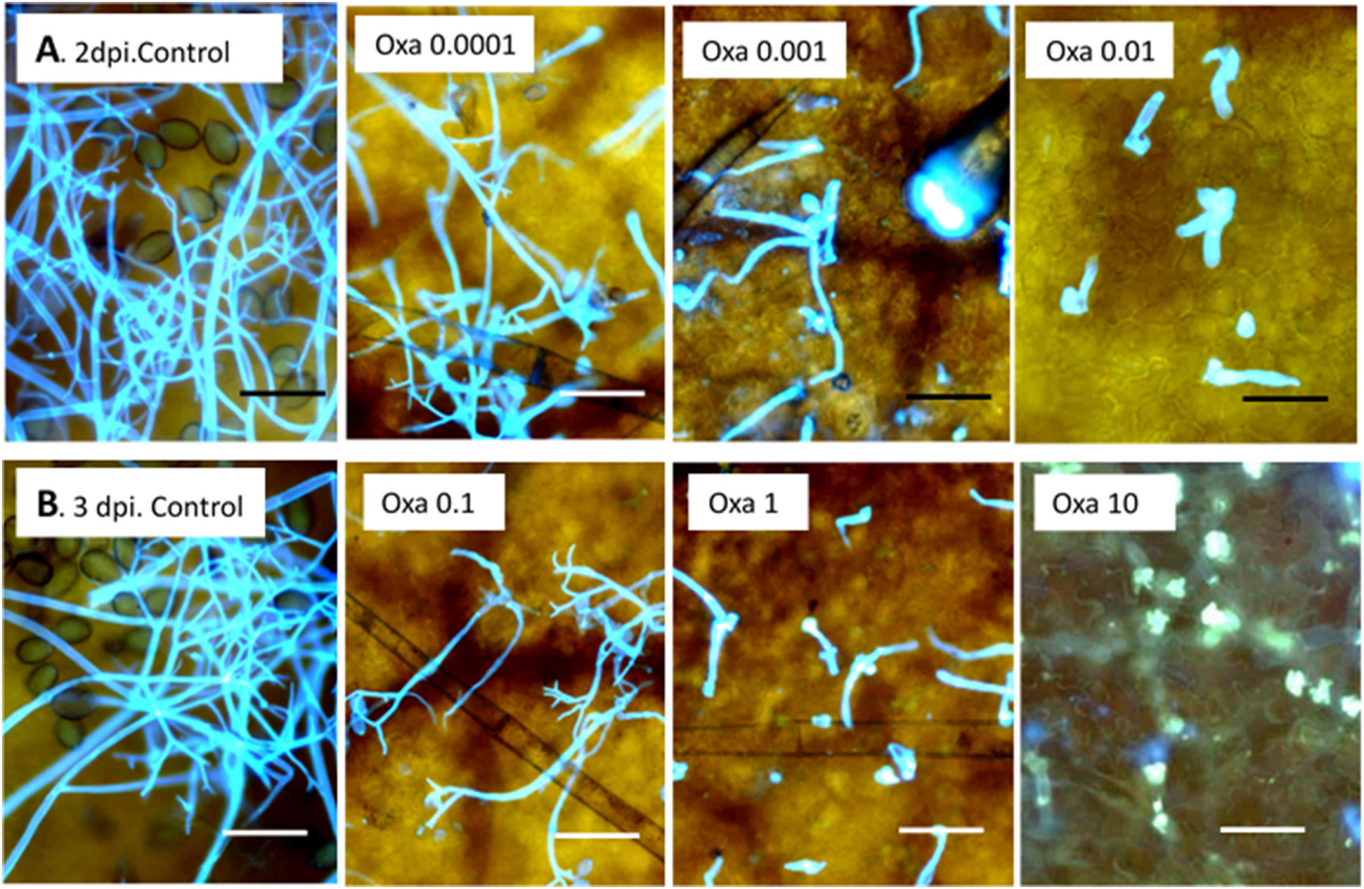
Fluorescence micrographs showing the suppression of sporulation of *P*. *cubensis* in cucumber leaves treated with oxathiapiprolin at 2 or 3dpi (calcofluor and basic aniline blue staining). **A**—Leaves treated with oxathiapiprolin of 0.0001–0.01 mg/l at 2 dpi. **B**—Leaves treated with oxathiapiprolin of 0.1–10 mg/l at 3 dpi. Photos were taken at 10 dpi. Bar = 50 μm. Note numerous sporangiophores and sporangia in the controls, few or deformed sporangiophores in treated leaves, and heavy callose encasement of haustoria at 10 mg/l.

Data in Fig 5A present lesion size in leaves treated with oxathiapiprolin at 0, 1, 2 or 3dpi. No lesions appeared at 0 dpi at ≥ 0.0001 mg/l oxathiapiprolin. At 1–3 dpi, suppression of lesion expansion was significantly stronger as the dose of the oomycide was higher. Higher doses were required at 3dpi compared to 2 or 1dpi (note the change in the dose values). Data in Fig 5B show that sporangiophore formation in such lesions was strongly suppressed. Thus, no sporangiophores were produced in leaves treated with the compound at 0 or 1dpi. Their number decreased significantly in a dose-dependent manner at 2 or 3dpi. The fluorescent micrographs presented in Fig 6A and 6B show that at increasing doses, oxathiapiprolin prevented sporangial formation, sporangiophore ramification or sporangiophore emergence from stomata.

**Fig 7:**
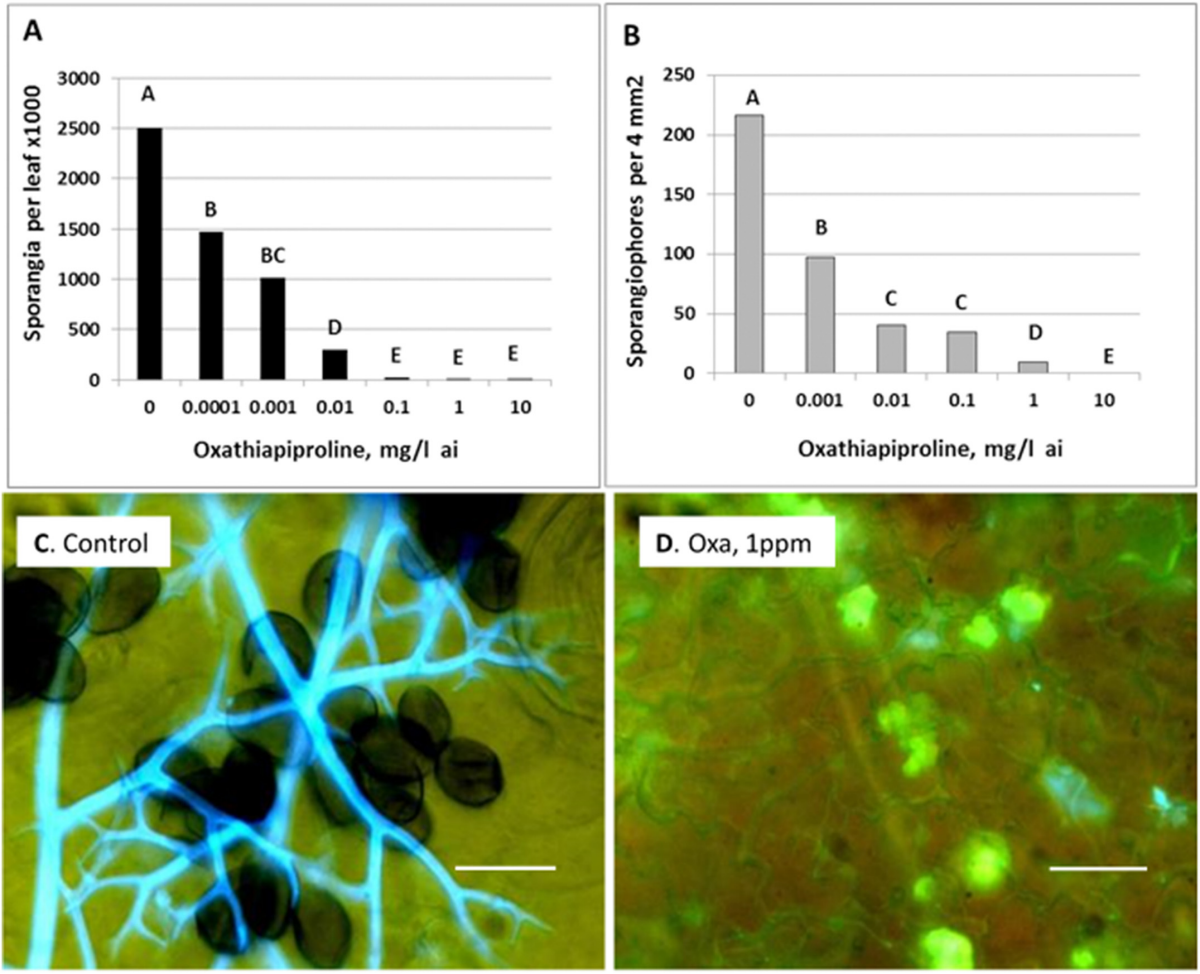
The effect of oxathiapiprolin on sporulation of *P*. *cubensis* in cucumber. **A**—Detached leaves were treated with oxathiapiprolin at 2dpi and examined for sporulation at 7 dpi. **B**—Detached leaves were treated with oxathiapiprolin at 5dpi and examined for sporulation at 7 dpi. Different letters on bars indicate on a significant difference at α = 0.05 (Tukey’s-Kramer HDS test). **C**- Sporulation of *P*. *cubensis* on an untreated control leaf of cucumber at 7 dpi. **D**—Encasement with callose of the houstoria of *P*. *cubensis* in a cucumber leaf treated with 1 mg/l oxathiapiprolin at 5dpi. Leaf discs were removed at 7 dpi and stained with basic aniline blue for callose (yellow) and calcofluor for cellulose (blue). Images were taken with an epifluorescent microscope under mixed UV-incandescent light illuminations. Bar = 30 μm. Note in **C**—the dark sporangia and blue sporangiophores, and in **D**—the callose-encased haustoria (yellow). Bar = 30 μm.

In the experiments shown in Fig 7, detached leaves were treated with oxathiapiprolin at 2 or 5 dpi and the numbers of sporangia (Fig 7A) or sporangiophores (Fig 7B) produced were counted at 7 dpi. Sporangial yield was reduced by 88, 99 and 99.8% in leaves treated at 2 dpi with oxathiapiprolin of 0.01, 0.1 and 1 mg/l, respectively (Fig 7A). The number of sporangiophores in leaves treated at 5dpi was reduced by 84, 95.4 and 100% with oxathiapiprolin of 0.1, 1 and 10 mg/l, respectively (Fig 7B). A visual illustration of such inhibition is given in the fluorescent micrographs presented in Fig 7C and 7D. While abundant sporangiophores and sporangia were produced in the control leaves (Fig 7C) no such organs were produced in leaves treated at 5 dpi with oxathiapiprolin of 1 mg/l (Fig 7D). Oxathiapiprolin induced heavy callose encasement of the haustoria colonizing the mesophyll which probably interferes with nutrients flow from the attacked host cells into the mycelia during sporogenesis.

#### Sporulation in naturally infected leaves

**Fig 8:**
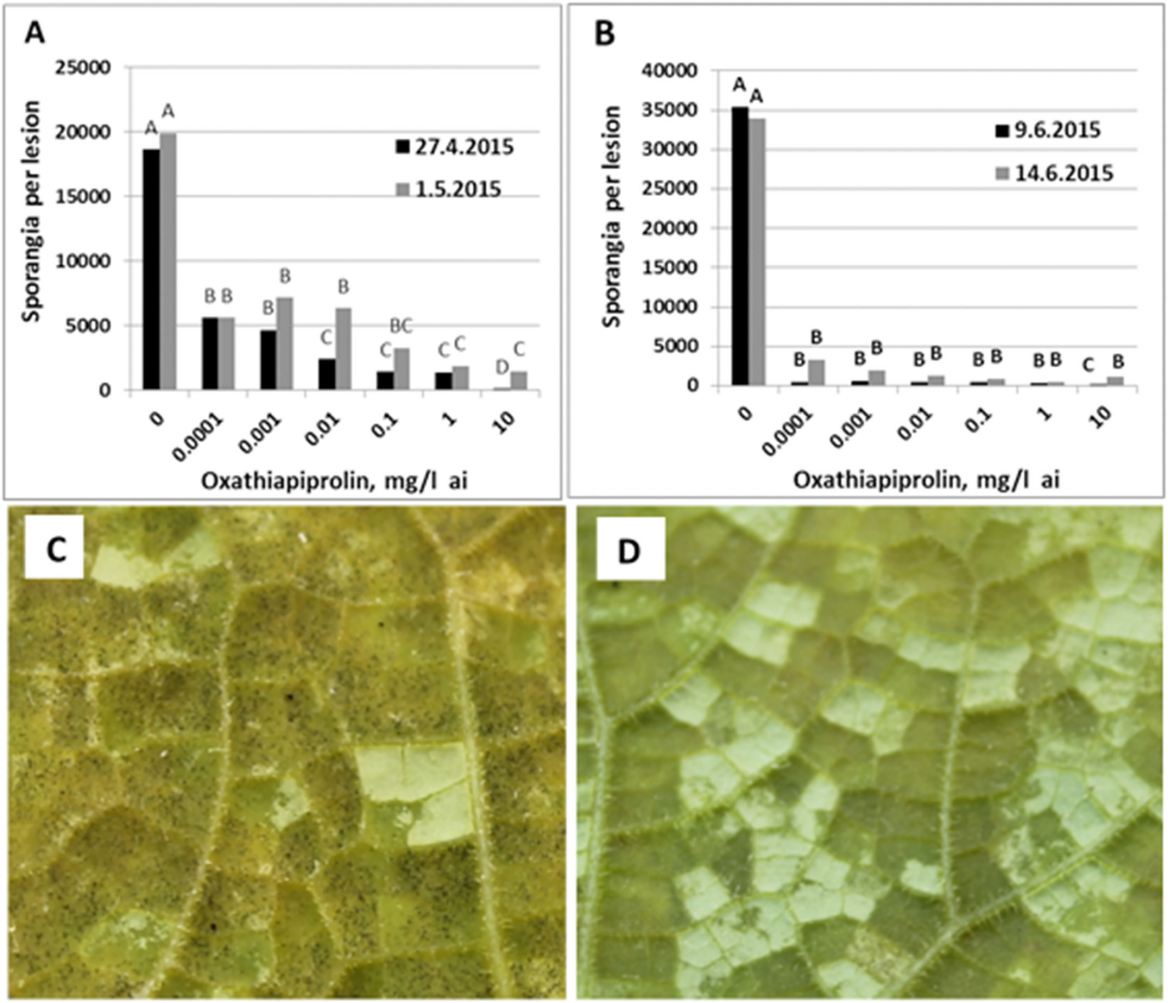
The effect of oxathiapiprolin on sporulation of *P*. *cubensis* in cucumber leaves naturally-infected with downy mildew. **A**—In leaves collected from Net-house 1 on April 27, 2015 and May 1, 2015. **B**—In leaves collected from Net-house 3 on June 9, 2015 and June 14, 2015. Different letters on bars indicate on a significant difference at α = 0.05 (Tukey’s-Kramer HDS test). **C**—Close up photo showing heavy sporulation in a control leaf. **D**—Close up photo showing no sporulation in a leaf treated with oxathiapirolin of 0.0001 mg/l on June 9. Photos were taken on June 10, 2015.

Oxathiapiprolin adversely affected sporulation in naturally-infected leaves of cucumber after were treated in the laboratory. Data presented in Fig 8 show 69–96% inhibition of sporulation in leaves collected from Net-house 1 on April 27, 2015 and May 1, 2015 (Fig 8A) and 95–99% inhibition in leaves collected from Net-house 3 on June 9, 2015 and June 14, 2015 (Fig 8B). A visual representation of such inhibition is provided in Fig 8C (control) and 8D (oxathiapirolin of 0.0001 mg/l applied June 9).

**Fig 9:**
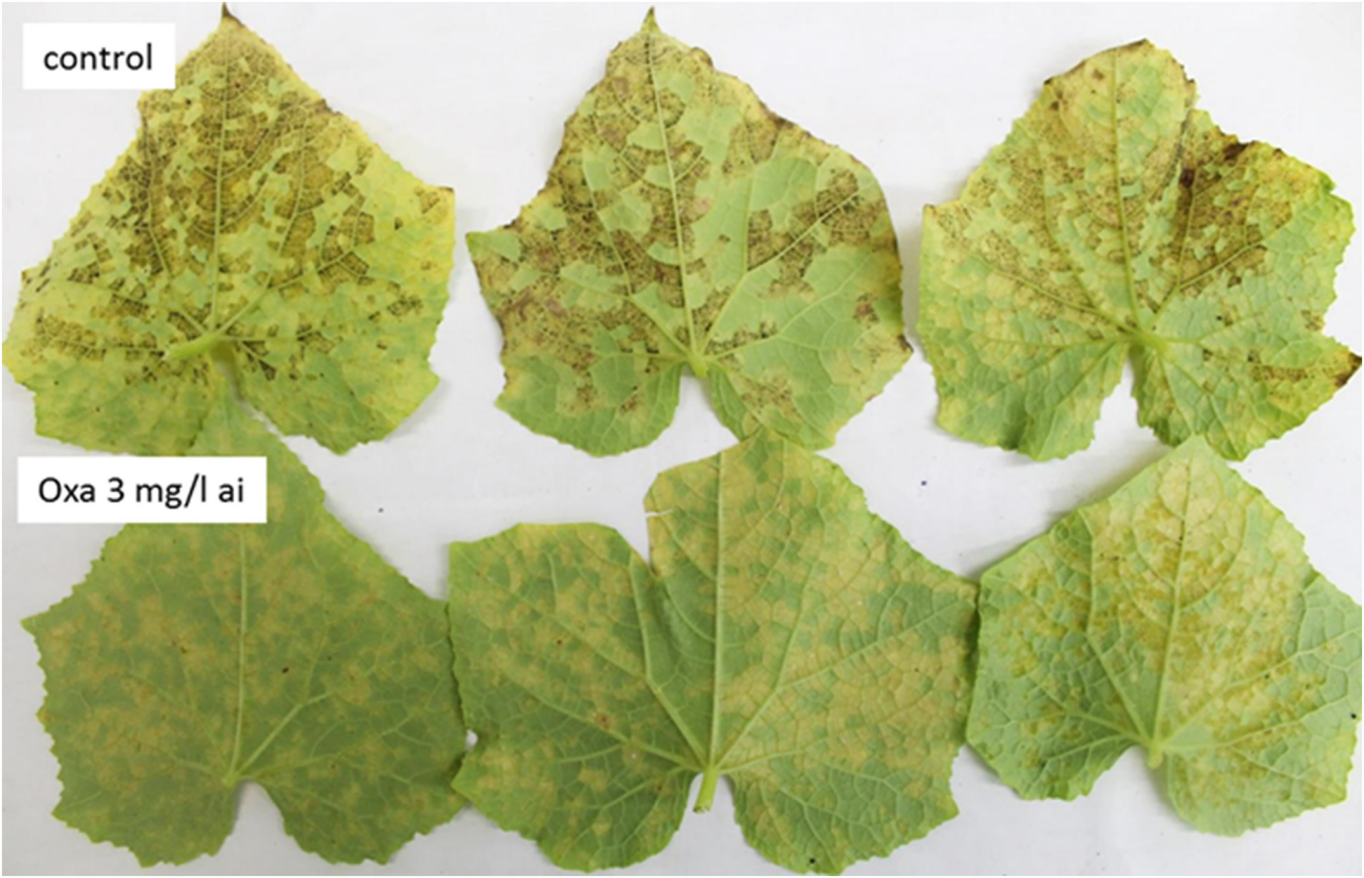
The effect of oxathiapiprolin on sporulation of *P*. *cubensis* in nature. Downy mildew-infected plants in Net-house 3 were sprayed with oxathiapiroplin of 3 mg/l ai at 8 am 11.6.2015, while control plants were left untreated. Leaves were collected (after a dewy night) at 8 am 12.6.2015 and photographed. Note the heavy sporulation in control leaves but no sporulation in treated leaves.

Oxathiapirolin of 3 mg/l strongly suppressed sporulation of *P*. *cubensis* when was applied (June 11, 2015) to downy mildew-infected cucumber plants in Net-house 3. Sporulation index (visual scale 0–3) in control leaves at one day after application ranged from 2–3 (2.75±0.35) as against 0–0.3 (0.08±0.10) in oxathiapirolin treated leaves (Fig 9).

#### Translaminar activity of oxathiapiprolin

**Fig 10:**
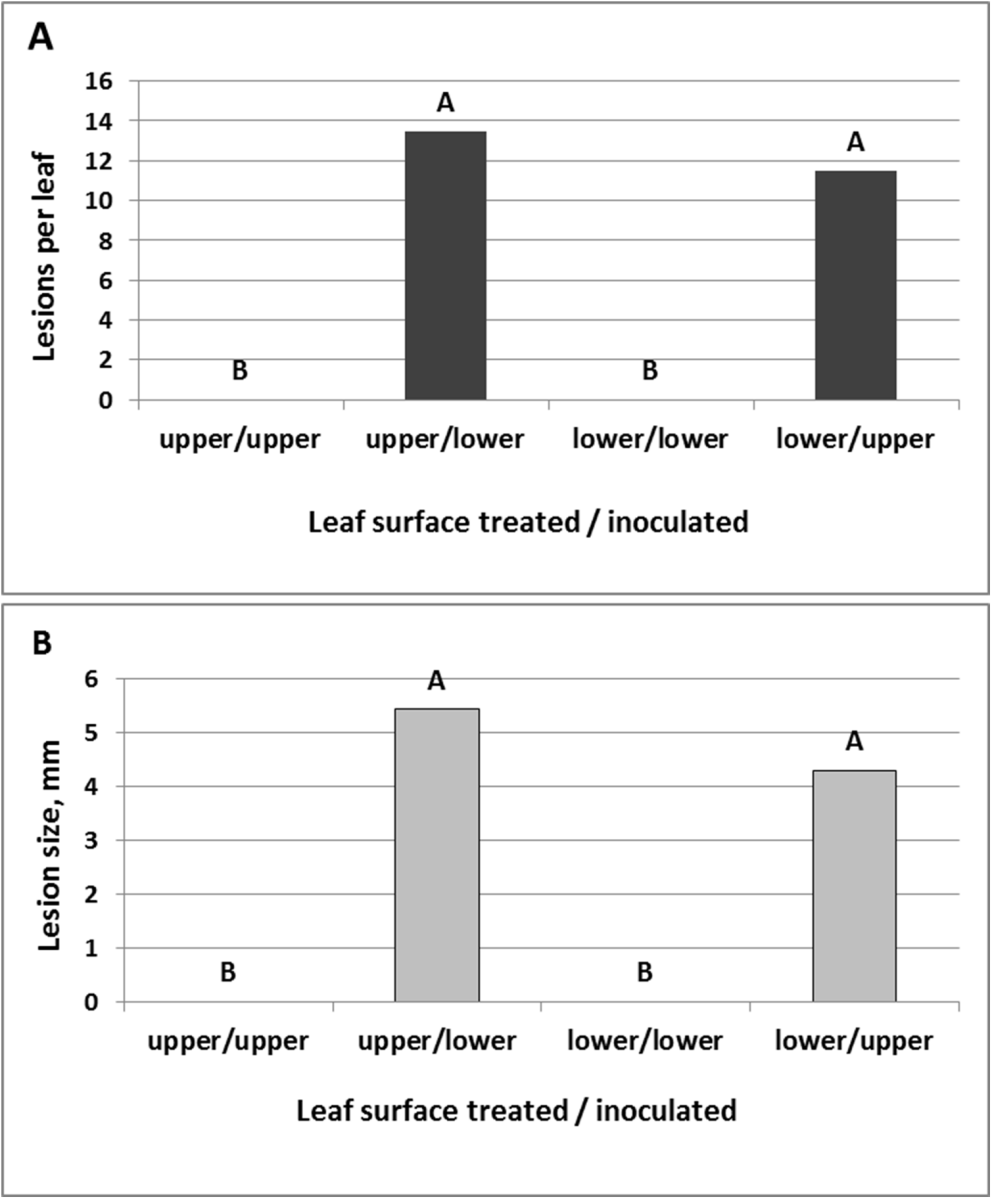
Translaminar control efficacy of oxathiapirolin against *P*. *cubensis* in detached cucumber leaves. The compound was sprayed at a concentration of 0.0001 mg/l on the lower or the upper leaf surface and leaves were inoculated with *P*. *cubensis* on the opposite leaf surface. Disease records were taken at 14 dpi. **A**—Lesions per leaf (out of 15 droplets inoculated). **B**—Lesion size, mm. Different letters on bars indicate on significant differences at α = 0.05 (Tukey’s-Kramer HSD-test).

Oxathiapiprolin exhibited translaminar properties: detached leaves sprayed with 0.0001–1 oxathiapiprolin mg/l and thereafter drop-inoculated with *P*. *cubensis* on the treated leaf surface (upper/upper or lower/lower) showed complete control of the disease at all doses at both 7 and 14dpi. Control inoculated leaves produced 15 lesions per leaf whose size ranged from 21–26 mm at 7 dpi and from 25–35 mm at 14dpi. Complete control of the disease was also seen in leaves treated with 0.001–1 mg/l, regardless of the surface treated/ inoculated (upper/lower, or lower/upper). However, at the lowest dose of 0.0001 mg/l, leaves treated/inoculated on opposite surfaces produced at 7dpi 8–10 lesions per leaf with a size of 4–4.5 mm and at 14dpi 11.5–13.5 lesions per leaf with a size of 4.3–5.4 mm (Fig 10), indicating on partial translaminar movement of the compound when applied at a low concentration.

#### Systemic translocation of oxathiapiprolin

**Fig 11:**
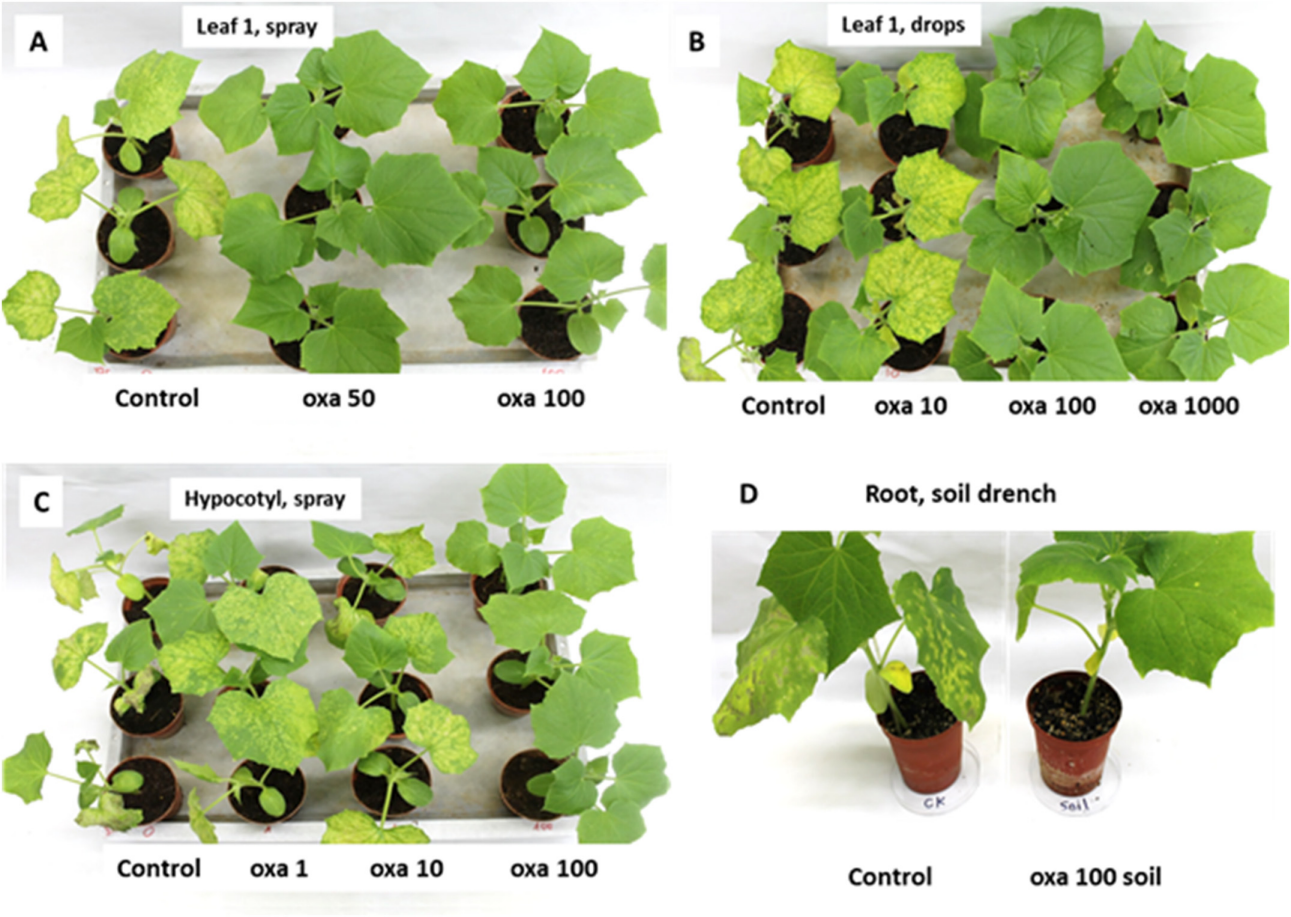
Translocation of oxathiapirolin from a treated organ to leaves. **A**—Leaf 1 (in 1-leaf plants) was treated by spray. **B**—Leaf 1 (in 1-leaf plants) was treated with 10 droplets of 25 μl each. **C**—hypocotyl of 2-leaf plants was treated by spray. **D**—Soil drench in 2 leaf plants. In **A** and **B** plants were inoculated after 4 days when leaf 2 has expanded. In **C** and **D** plants were inoculate one day after treatment. Photographs were taken at 7dpi.

Oxathiapiprolin exhibited acropetal systemic mobility in cucumber plants. When sprayed at 50 or 100 mg/l to leaf 1, both leaf 1 and the newly-developed leaf 2 were fully protected against downy mildew whereas the control untreated inoculated plants developed 260±50 lesions per plant (Fig 11A). When droplets containing 10 mg/l were applied to leaf 1, the treated leaf 1 was fully protected but leaf 2 showed no protection (Fig 11B). However, application of droplets containing 100 or 1000 mg/l oxathiapiprolin were fully protective to both leaf 1 and leaf 2 (Fig 11B). A spray of 1 or 10 mg/l applied to the hypocotyl resulted with no protection of the leaves relative to the control plants but a spray of 100 mg/l provided full protection against the disease (Fig 11C). Oxathiapiprolin applied as a soil drench to the root system (1 ml, 100 mg/l) supplied full protection against the disease (Fig 11D).

#### Seed coating

Seed coating had no impact on percent germination or growth rate of cucumber plants compared to uncoated seeds. Downy mildew development was dramatically suppressed in plants developed from coated seeds relative to plants developed from regular seeds. Number of lesions per plant in control untreated-inoculated plants at the 1-leaf, 2-leaf and 3-leaf stage was 70±25, 180±54 and 375±28, respectively as against 0, 0 and 1.0±1.7 in coated-seed plants, respectively. All lesions in 3-leaf plants developed in leaf 3; no lesions developed in leaves 1 or 2.

## Discussion

Management of downy mildew in cucurbits relies mainly on oomycidesdeployment. Disease outbreaks became more frequent and more severe since 2002 in Israel [33,36], 2004 in the USA [4,37] and 2009 in Europe [1]. The appearance of new aggressive pathotypes with expanded host range and mating capability seems to be responsible for this resurgence of *P*. *cubensis* [1,36,38]. In the USA, the long-standing genetic resistance in cucumber provided by the *dm-1* gene became no longer effective in 2004, probably due to the migration of new pathotypes via seeds [39]. In many countries, including Israel, the pathogen developed resistance against phenylamides (e.g. metalaxyl, mefenoxam), carboxyl acid amides (CAAs, e.g. dimethomorph, mandipropamid) and strobilurins (e.g. azoxystrobin) [5–10,13,19–21,40,41].

Oxathiapiprolin is a new oomycide, with a novel mode of action. As shown here, it is capable of controlling isolates of *P*. *cubensis* resistant PA, QoI and CAAs. We have examined the inhibitory effects of oxathiapiprolin on almost every stage in the asexual life cycle of *P*. *cubensis*. Oxathiapiprolin allowed the cleavage of the zoospores inside the sporangium during zoosporogenesis (not shown) but suppressed their discharge from the sporangium. Similar inhibition of zoospore release was observed with *P*. *capsici* [30]. Inhibition of zoospore release was almost complete both *in vitro* and *in vivo* on leaf surface. When added to motile zoospores *in vitro* oxathiapiprolin had a weak negative effect on the speed or duration of their motility, suggesting that it does not hamper the energy supply required by the locomotive machinery of the zoospore. Oxathiapiprolin enhanced the disintegration of zoospores *in vitro* and *in vivo*, causing rupture of their membrane. No quantitative figures are available on this aspect.

The most pronounced effect of oxathiapiprolin was its inhibitory effect on cystospore germination. Very low doses of the compound were required to completely avoid germination of the cystospores either *in vitro* or *in vivo*. Similar inhibition of cystospore germination was observed *in vitro* with *P*. *capsici* [29,30]. The biochemical mechanism(s) responsible for this inhibition is not known. CAA fungicides, which target the cellulose synthase CesA3 protein of oomycetes [19] are also strong inhibitors of cystospore germination but the dose required is x100 higher compared to oxathiapiprolin [42].

Oxathiapiprolin targets oxysterol-binding proteins in oomycetes [25]. Oxysterol-binding protein (OSBP)-related proteins (ORPs) are lipid-binding proteins implicated in many cellular processes including signaling, vesicular trafficking, lipid metabolism, and non-vesicular sterol transport [43]. It might occur that oxathiapiprolin affects cellulose micro-fibrils trafficking towards the tip membrane of the germinating cystospore, thus preventing germ-tube emergence.

The effect of oxathiapiprolin on downy mildew development was strongly dependent on the time of its application relative to the inoculation event. When applied preventively before inoculation, oxathiapirolin totally prevented disease production whereas when applied after inoculation it enabled disease development but reduced lesion expansion and sporangiophore/sporangia production, probably due to the inhibition of mycelium growth.

Because cystospore germination is inhibited *in vivo*, no stomatal penetration could take place and no lesions of downy mildew were produced in leaves treated preventively with oxathiapiprolin. All 26 isolates used in this study exhibit similar high sensitivity to the compound, regardless of the host used for inoculation, suggesting on high expected efficacy against a variety of pathotypes known to prevail in various locations around the globe [4,33]. Very low doses of the compound were required to avoid lesion formation, the lowest dose known in the oomycides world. This is a great advantage of this oomycide but at the same time may turn it into a high-risk oomycide in terms of resistance.

Oxathiapiprolin possess curative effects against *P*. *cubensis*. When applied to already-infected leaf tissue it induces callose encasement of the haustoria. Callose encasement of haustoria was implicated in genetic resistance of melon against *P*. *cubensis* [35] and in resistance induced by DL-β amino-butyric acid (BABA) against *Bremia lactucae* in lettuce [44]. Such encasement may starve the pathogen, lead to cessation of mycelium growth and restrict lesion expansion. Lesion expansion arrest was also reported for metalaxyl, but it lasted for only one or two days after inoculation [45] as compared to 3 days with oxathiapiprolin.

Oxathiapiprolin also exhibited anti-sporulative effect against *P*. *cubensis*. When applied to artificially inoculated leaves at the terminal phase of disease development it strongly suppressed sporangiophore emergence from stomata and therefore no sporangia were formed. When applied in the laboratory to naturally-infected leaves a strong suppression of sporangial production was obtained with 0.0001 mg/l of the compound. Infected plants treated in the field with 3 mg/l of oxathiapiprolin failed to sporulate, suggesting a “knock-down” effect of the compound. A strong anti-sporulation activity of oxathiapiprolin was reported for *P*. *capsici in vitro* [29,30]. Other oomycides (e.g. phenylamides, CAAs) exhibit anti-sporulation effects but the doses required are higher compared to oxathiapiprolin [42,45].

Oxathiapiprolin is shown here to translocate acropetally in cucumber plants. When applied to the root system or to the hypocotyl, leaves were protected against downy mildew. When was applied to leaf 1, leaf 2 was protected. However, relatively high doses were required to achieve such protection (50–100 mg/l) indicating on partial mobility (or degradation) of oxathiapiprolin in the plant. Systemic protection by soil drench was also observed in potted tomato plants against late blight and in basil plants against downy mildew (Y. Cohen, *unpublished data*). Soil application of oxathiapiprolin was effective against *P*. *nicotianae* in tobacco [46].

Oxathiapiprolin exhibited excellent activity against *P*. *cubensis* when applied as seed dressing (~40 μg per seed). Plants developed from such seeds were fully protected against downy mildew at the 1, 2, and 3 true leaf stage, suggesting on effective mobility from the embryo to the developing seedling. Using radiolabeled oxathiapiprolin may provide better insight on the uptake, mobility and degradation of the compound in the plant (e.g. see [47] for ^14^C-cymoxanil). Oxathiapiprolin applied as seed treatment was already reported to control downy mildew in basil [27].

Oxathiapiprolin exhibits remarkable translaminar movement across the leaf lamina. At concentration of ≥ 0.001 mg/l disease was completely controlled regardless of whether the inoculum was applied to the treated or the untreated leaf surface. Only at the lowest dose used of 0.0001 mg/l one could distinguished that translaminar movement of the compound was partial.

In conclusion: oxathiapiprolin, a novel oomycide with a new mode of action, is highly effective in controlling downy mildew in cucurbits caused by *P*. *cubensis*. It was effective against various pathotypes, mating types, and oomycide-resistant isolates. It is effective against all stages in the asexual life cycle of the disease. It possesses preventive and curative properties. It shows excellent translaminar mobility and partial acropetal systemicity.

The availability of oxathiapiprolin to the farmers may now improve significantly their ability to combat this devastating downy mildew disease of cucurbits. To avoid possible resistance build up in *P*.*cubensis*, oxathiapiprolin should be mixed with unrelated oomycide (s) before applied in the field.

## Supporting Information

S1 File Efficacy of oxathiapiprolin applied via the root system. (XLSX)

S2 File Efficacy of oxathiapiprolin applied to various cucurbit species (XLSX)

S3 File Translaminar movement of oxathiapiprolin. (XLSX)

S4 File Effect of oxathiapiprolin on zoospore release. (XLSX)

S5 File Curative efficacy of oxathiapiprolin. (XLSX)

S6 File Preventive vs curative efficacy of oxathiapiprolin. (XLSX)

S7 File Efficacy of oxathiapiprolin against sporophore production. (XLSX)

S8 File Efficacy of oxathiapiprolin against sporulation. (XLSX)

S9 File Efficacy of oxathiapiprolin in intact plants (XLSX)

S10 File Curative efficacy of oxathiapiprolin at 2 and 4 dpi. (XLSX)
